# Physical Fitness Tests and Type 2 Diabetes Among Japanese: A Longitudinal Study From the Niigata Wellness Study

**DOI:** 10.2188/jea.JE20170280

**Published:** 2019-04-05

**Authors:** Haruki Momma, Susumu S Sawada, Kiminori Kato, Yuko Gando, Ryoko Kawakami, Motohiko Miyachi, Cong Huang, Ryoichi Nagatomi, Minoru Tashiro, Masahiro Ishizawa, Satoru Kodama, Midori Iwanaga, Kazuya Fujihara, Hirohito Sone

**Affiliations:** 1 Division of Biomedical Engineering for Health and Welfare, Tohoku University Graduate School of Biomedical Engineering, Miyagi, Japan; 2 Department of Health Promotion and Exercise, National Institutes of Biomedical Innovation, Health and Nutrition, Tokyo, Japan; 3 Department of Hematology, Endocrinology and Metabolism, Niigata University Faculty of Medicine, Niigata, Japan; 4 Department of Laboratory Medicine and Clinical Epidemiology for Prevention of Noncommunicable Diseases, Niigata University Graduate School of Medical and Dental Sciences, Niigata, Japan; 5 Faculty of Sport Sciences, Waseda University, Saitama, Japan; 6 Department of Physical Education and Sports Science, Zhejiang University, Zhejiang, China; 7 Department of Medicine and Science in Sports and Exercise, Tohoku University Graduate School of Medicine, Miyagi, Japan; 8 Niigata Association of Occupational Health, Niigata, Japan

**Keywords:** grip strength, balance, discrete-time logit model, hyperglycemia

## Abstract

**Background:**

Simple physical fitness test can be a useful potential predictor of type 2 diabetes (T2DM). We examined the association between performances on simple physical fitness tests and the incidence of T2DM.

**Methods:**

This longitudinal study was conducted in 21,802 nondiabetic Japanese (6,649 women) aged 20 to 92 years, who underwent all physical fitness tests at baseline (April 2001 to March 2002). From April 2001 to March 2008, physical fitness tests, including grip strength, vertical jump, single-leg balance with eyes closed, forward bending, whole-body reaction time, and supine legs-up, were performed every year. Participants had physical fitness tests at least two times during the period. T2DM was also annually determined based on fasting blood glucose, glycated hemoglobin, and self-reported diabetes during the period. Discrete-time logit models were used to examine the influence of the serial level of each physical fitness test on the incidence of T2DM.

**Results:**

During the entire study period, 972 participants developed diabetes. Lower relative grip strength (grip strength/body weight) and single-leg balance performance were associated with a higher incidence of T2DM. For relative grip strength, as compared with the fourth quartile group, the odds ratios for other groups ranged from 1.16 to 1.56 (*P* for trend < 0.001). For single-leg balance, the odds ratios ranged from 1.03 to 1.49 (*P* for trend < 0.001).

**Conclusion:**

The performance of a simple single-leg balance test as well as that of a grip strength test were negatively associated with the risk of T2DM among Japanese.

## INTRODUCTION

The prevalence of type 2 diabetes mellitus (T2DM) has been increasing worldwide.^1^ The number of individuals with diabetes in the world is predicted to increase to 642 million by 2040 if effective strategies for diabetes prevention are not developed.^1^ Given that the prevalence of T2DM is a serious issue, particularly in low-income countries—including those of Asia,^2^ where blood tests are not always easy to conduct—the identification of simple, non-invasive, and useful predictors of T2DM is desirable for early detection and prevention in both clinical and preclinical settings.

Regular exposure to exercise and physical activity results in a variety of adaptations in the body, including skeletal muscle tissue. Because these changes have protective effects on the development of T2DM,^3^^,^^4^ regular exercise and physical activity are known as key elements in the prevention of T2DM. Given that improved physical fitness represents adaptation to regular exercise and physical activity, the measurement of physical fitness can be useful to predict the incidence of T2DM. Previous studies showed that cardiorespiratory fitness, a component of physical fitness, was negatively associated with a lower risk of T2DM.^5^^,^^6^ However, cardiorespiratory fitness is not readily assessed in clinical and preclinical settings,^7^ due to the complexity of the measurements. Recently, grip strength, an indicator of muscle strength, has been considered as a quick and inexpensive predictor of T2DM,^8^^–^^13^ although some studies have reported no association between grip strength and T2DM.^10^^–^^13^ Therefore, whether grip strength is associated with the risk of T2DM remains controversial. In addition, physical fitness also includes other components, such as muscular endurance, power, balance, reaction time, and flexibility,^14^^,^^15^ and each component of fitness is not necessarily independent. Moreover, some of the measurements for these components are similar in their simplicity to that of grip strength.

Therefore, the purpose of this study was to examine the association between various components of physical fitness and the incidence of T2DM in a Japanese population. In this study, we considered time-varying physical fitness levels, because physical fitness changes with aging, lifestyle, and physical condition.

## METHODS

### Participants

Our study included Japanese individuals who underwent annual health examinations for health screening by the Niigata Association of Occupational Health in Niigata, Japan. Details on the health examinations by the Niigata Association of Occupational Health have been provided elsewhere.^16^^,^^17^

In this longitudinal study, we included 55,347 Japanese individuals (19,377 women, 35.0%) who had an initial health examination from April 2001 through March 2002. Individuals (*n* = 21,209) who did not undergo all physical fitness tests at baseline were excluded. Furthermore, to obtain time-varying physical fitness levels, individuals who did not have at least two physical fitness tests from April 2002 through March 2008 were also excluded (*n* = 10,548). In addition, individuals who had no information on blood glucose or glycated hemoglobin (HbA1c) levels (*n* = 11) or had diabetes (*n* = 1,315) at baseline were excluded. We also excluded individuals with a history of cancer (*n* = 194) or cardiovascular disease (*n* = 268). Finally, 21,802 individuals (6,649 women) aged 20–92 years were eligible for the analysis.

This study was approved by the ethics committees of the National Institutes of Biomedical Innovation, Health and Nutrition and the School of Medicine, Faculty of Medicine, Niigata University. We obtained written informed consent from each individual.

### Assessment of clinical variables

A questionnaire was used to assess smoking, alcohol, breakfast and physical activity habits, and history of dyslipidemia, hypertension, and diabetes at each annual examination. Height and weight were measured without shoes or heavy clothing, and body mass index (BMI) was calculated (kg/m^2^). Blood pressure was measured with the participant in a sitting position. Blood samples were collected after an overnight fast and measurements were made using automatic clinical chemistry analyzers (HITACHI 7250, 7600, and 7700; Hitachi, Tokyo, Japan) for triglyceride, low-density lipoprotein (LDL)-, and high-density lipoprotein (HDL)-cholesterol. Blood glucose and HbA1c levels were determined using an automated analyzer (JCA-BM9030; JEOL, Tokyo, Japan). The HbA1c value was converted to the National Glycohemoglobin Standardization Program equivalent value (%) using the following formula: HbA1c = 1.02 × HbA1c (Japan Diabetes Society) + 0.25.^18^

### Physical fitness tests

Physical fitness tests were conducted after a warm-up exercise at the time of the health examinations. The details of physical fitness tests are described in eMaterial 1.

Muscle strength was measured using a grip strength dynamometer (T.K.K. 5401; Takei Scientific Instruments Co., Ltd, Niigata, Japan) with the individuals in a standing position. Grip strength was measured once for each hand alternately. The highest value was used and the relative grip strength (grip strength [kg]/body weight [kg]) was calculated, because grip strength is influenced by body size.^19^^,^^20^ Lower extremity muscle power was assessed using a vertical jump-measuring instrument (T.K.K. 5414; Takei Scientific Instruments), by measuring the time that the individual had both feet off the ground. Each individual performed two trials, and the best performance was used. Because the vertical jump is also influenced by body size, we calculated relative vertical jump (vertical jump [cm]/body weight [kg]). Static balance was assessed by measuring the duration (s) of single-leg balance with eyes closed using a stopwatch. Participants were asked to keep standing on a firm surface for 240 s with their hands placed on their hips. Participants performed the test a maximum of 3 trials, and the best value was used. Trunk flexibility was measured using a digital flexibility testing device (T.K.K. 5403; Takei Scientific Instruments). Participants were asked to stand on a measuring bench, placing the toes even with the front edge of the bench, and then asked to bend over and reach down as far as possible without bouncing, while keeping the knees locked. Performance was scored as the distance (cm) reached by the middle fingers. Participants performed a single trial of the flexibility test. Whole-body reaction time was measured using a pressure-sensing mat (T.K.K. 5408; Takei Scientific Instruments). Participants were asked to stand on the mat switch and to jump upright as quickly as possible in response to a light sign that was 2 m away from the mat. The time (ms) between the flashing and the disappearance of foot pressure from the mat was measured. Participants underwent three trials and the average of the three trials was calculated. A supine legs-up test was used to measure the muscular endurance of the lower abdominal muscles. The participants were asked to raise both extended legs from the floor approximately 30 cm and hold this position as long as possible. The time (s) was recorded by a stopwatch and the test was ended when the participants were no longer able to maintain legs clearance. Participants performed a single trial of the legs-up test.

### T2DM

T2DM was defined by a fasting glucose level of ≥126 mg/dL (7.0 mmol/L), HbA1c ≥48 mmol/mol (6.5%), or a self-reported history of previously diagnosed diabetes or current medication for diabetes.^21^ Individuals without diabetes at baseline were considered to have incident T2DM when they met any of these conditions in the subsequent health checkups from April 2002 through March 2008.

### Statistical analysis

Multiple imputation with chained equations was used to handle missing data in this study. Analysis of imputed datasets reduces the effects of potential biases introduced by missing data.^22^^,^^23^ The percentage of missing data for each physical fitness measurement during the entire study period in 114,542 observations ranged from 13.8% for grip strength to 17.9% for the legs-up test. The missing values for covariates fell within 0.1% of the observations, while the information on habitual physical activity was missing for 45.9%. These missing values were imputed according to a model comprising all variables in the analytic model described below and habitual physical activity. We entered habitual physical activity only into the model of imputation, not into the analytic model, because of the large number of missing data. We created 10 multiple imputed datasets and showed pooled estimates among these datasets.

Discrete-time logit models were used to investigate the associations between physical fitness and the incidence of diabetes.^24^^,^^25^ These models were used in previous studies.^26^^–^^28^ This analysis has important advantages compared with time-to-event survival analysis (ie, the Cox proportional hazards model), because it does not require an assumption of proportional hazards and allows greater flexibility in modeling time-varying covariates. In this approach, each discrete time unit for each individual is treated as a separate observation or unit of analysis (ie, person-period dataset). For each of these observations, the outcome variable is coded 1 if an event (ie, diabetes in this study) occurred to that individual in that time unit; otherwise, it is coded zero. Therefore, if an individual developed diabetes at time 7 (April 2007 through March 2008), seven different observations were obtained. For the seventh observation, the outcome variable was coded 1. For the other six observations, the outcome variable was coded zero. If an individual did not develop diabetes until the end of the follow-up period, the seven different observations were coded as zero. If an individual developed diabetes at time 4 (April 2004 through March 2005), only four different observations were obtained. Similarly, when an individual was censored at time 4, four different observations were obtained with the all outcome variables coded as zero. We used 1-year intervals (from April through March) rather than assuming continuous time for the following reasons: (1) we assessed whether T2DM developed at any time within each time interval, and (2) the entire study period was relatively short (within 7 years). The exposure variables for each of these observations were assigned whatever values they had at that particular unit of time. In this study, all variables except for sex in the analyses were used as time-varying covariates. For the analysis of relative grip strength, individuals were first divided into quartiles (Q1, Q2, Q3, and Q4) based on the relative grip strength at baseline (time 1). Then, they were also categorized into quartiles based on the performance of grip strength at time 2 to 7, respectively. This categorization was applied to other physical fitness and conducted after the stratification by sex and age (in 5-year increments), because physical fitness was influenced by sex and age. Therefore, the variables for physical fitness for each observation were coded as the level of quartiles (1, 2, 3, and 4) at that particular unit of time. For the legs-up test, because more than half of the participants could keep their legs up for 90 s, participants were divided into 2 groups based on a cutoff of 90 s every year.

We applied the logistic regression to our person-period dataset in order to examine the influence of serial physical fitness on the development of T2DM.^24^^,^^25^ We adjusted for potential confounders including age (continuous variable), sex (man or woman), smoking (never, former, or current), alcohol (never, 1–2 days/week, 3–6 days/week, or every day), skipping breakfast (no or yes), dyslipidemia (defined as triglycerides ≥150 mg/dL, LDL-cholesterol ≥140 mg/dL, HDL-cholesterol <40 mg/dL, or self-reported history clinician-diagnosed dyslipidemia [no or yes]), and hypertension (defined as systolic blood pressure ≥140 mm Hg, diastolic blood pressure ≥90 mm Hg, or self-reported history clinician-diagnosed hypertension [no or yes]) (model 1). In addition to model 1, model 2 included BMI (<18.5, ≥18.5 and <25.0, ≥25.0 and <30.0, or ≥30), because we assumed that not only obesity but also underweight might have an influence on the incidence of diabetes.^29^^,^^30^ Furthermore, to explore potential effect modifications, we examined interactions between each physical fitness measurement and covariates by adding cross-product terms to model 3.

We conducted several sensitivity analyses. First, to eliminate the influence of possible preexisting diabetes at baseline, we excluded participants who developed diabetes within 2 years after the start of follow up. Second, we performed complete-case analysis (*n* = 5,335) using the above-mentioned models to check the robustness of the results. In addition, using the complete-cases dataset, we also conducted the same analysis with 1-year “lagged” physical fitness values and covariates against diabetes. For grip strength, we used absolute strength instead of relative strength to be comparable to results from previous studies. Finally, we adjusted for the mutual physical fitness values as continuous variables in addition to model 2.

For statistical analysis, all analyses were done using SPSS version 22 (IBM Japan, Tokyo, Japan).

## RESULTS

### Study participants

The median age of the participants at baseline was 50 (interquartile range, 44–46) year. The median follow-up period was 5 year. During the follow-up period from April 2002 through March 2008, 972 (4.5%) participants developed diabetes. The baseline characteristics of the participants are shown in Table 1. eTable 1 compares the characteristics of participants included in and excluded from the analysis. The percentage of women among the included participants was 7.4% higher than that among the excluded participants. Although there were differences among all the variables, except for single-leg balance, these negligible differences were thought to be due to the large sample size.

**Table 1.** Baseline characteristics of participants

	Total (*n* = 21,802)	Men (*n* = 15,153)	Women (*n* = 6,649)
Age, years	50.0 (44.0, 56.0)	50.0 (43.0, 56.0)	50.0 (44.0, 56.0)
Height, cm	164.9 (158.2, 170.6)	168.2 (163.9, 172.5)	155.4 (151.6, 159.2)
Weight, kg	61.4 (54.2, 68.5)	65.1 (59.3, 71.2)	52.4 (48.0, 57.3)
BMI, kg/m^2^	22.7 (20.9, 24.5)	23.1 (21.3, 24.9)	21.7 (20.0, 23.6)
SBP, mm Hg	117.0 (108.0, 127.0)	119.0 (110.0, 129.0)	112.0 (104.0, 122.0)
DBP, mm Hg	76.0 (68.0, 83.0)	78.0 (70.0, 84.0)	70.0 (65.0, 79.0)
TG, mg/dL	96.0 (67.0, 139.0)	108.0 (77.0, 156.0)	73.0 (55.0, 102.0)
LDL-C, mg/dL	117.0 (98.0, 139.0)	118.0 (99.0, 139.0)	116.0 (97.0, 138.0)
HDL-C, mg/dL	59.0 (50.0, 71.0)	56.0 (48.0, 67.0)	67.0 (57.0, 77.0)
TC, mg/dL	202.0 (181.0, 224.0)	201.0 (180.0, 223.0)	204.0 (183.0, 228.0)
Blood glucose, mg/dL	93.0 (88.0, 100.0)	95.0 (89.0, 101.0)	90.0 (86.0, 96.0)
HbA1c, mmol/mol	32.2 (30.1, 35.5)	32.2 (30.1, 35.5)	33.3 (30.1, 36.6)
HbA1c, %	5.1 (4.9, 5.4)	5.1 (4.9, 5.4)	5.2 (4.9, 5.5)
Smoking status, *n* (%)^a^			
Never smoker	10,034 (46.0)	4,130 (27.3)	5,904 (88.8)
Former smoker	4,290 (19.7)	4,085 (27.0)	205 (3.1)
Current smoker	7,416 (34.0)	6,901 (45.5)	515 (7.7)
Missing data	62 (0.3)	37 (0.2)	25 (0.4)
Drinking status, *n* (%)^a^			
None	5,496 (25.2)	1,786 (11.8)	3,710 (55.8)
1–2 days per week	3,442 (15.8)	2,074 (13.7)	1,368 (20.6)
3–6 days per week	5,099 (23.4)	4,121 (27.2)	978 (14.7)
7 days per week	7,704 (35.3)	7,135 (47.1)	569 (8.6)
Missing data	61 (0.3)	37 (0.2)	24 (0.4)
Skipping breakfast, *n* (%)			
No	20,470 (93.9)	14,104 (93.1)	6,366 (95.7)
Yes	1,271 (5.8)	1,012 (6.7)	259 (3.9)
Missing data	61 (0.3)	37 (0.2)	24 (0.4)
Exercise habit, *n* (%)			
No	9,497 (43.6)	6,590 (43.5)	2,907 (43.7)
Yes	6,255 (28.7)	4,246 (28.0)	2,009 (30.2)
Missing data	6,050 (27.7)	4,317 (28.5)	1,733 (26.1)
Hypertension, *n* (%)	4,263 (19.6)	3,418 (22.6)	845 (12.7)
Dyslipidemia, *n* (%)	9,130 (41.9)	6,959 (45.9)	2,171 (32.7)
Relative grip strength, kg/kg	0.63 (0.54, 0.71)	0.67 (0.61, 0.74)	0.51 (0.45, 0.57)
Relative vertical jump, cm/kg	0.66 (0.57, 0.76)	0.69 (0.60, 0.78)	0.61 (0.52, 0.70)
Single-leg balance, s	35.0 (16.0, 66.0)	34.0 (16.0, 64.0)	37.0 (16.0, 74.0)
Forward bend, cm	8.0 (3.0, 13.0)	6.0 (1.0, 11.0)	12.0 (7.5, 16.0)
Reaction time, ms	349.0 (323.0, 383.0)	342.0 (317.0, 372.0)	370.0 (340.0, 408.0)
Legs-up, s	90.0 (72.0, 90.0)	90.0 (90.0, 90.0)	78.0 (49.0, 90.0)

BMI, body mass index, calculated as weight in kilograms divided by height in meters squared; HbA1c, hemoglobin A1c; HDL-C, high-density lipoprotein cholesterol; IQR, interquartile range; LDL-C, low-density lipoprotein cholesterol; SBP, systolic blood pressure; DBP, diastolic blood pressure; TC, total cholesterol; TG, triglyceride.

Data are expressed as medians (interquartile range) for continuous variable and number (percentage) for categorical variable.

^a^Percentages and numbers of men and women may not sum to 100 or total numbers due to rounding.

### Physical fitness and T2DM

Table 2 shows the odds ratios (ORs) and 95% confidence intervals (CIs) of the incidence of T2DM among the quartiles of each physical fitness measurement. Because there was no interaction between any physical fitness measurement and sex, we presented combined results for men and women. For relative grip strength, when comparing the fourth quartile (Q4) with the other quartiles (Q3, Q2, and Q1), the ORs were higher in quartiles with higher relative grip strength (*P* for trend < 0.001). This negative relationship remained after adjusting for confounders in model 1 (*P* for trend < 0.001). Although additional adjustment for BMI (model 2) attenuated this relationship, the negative relationship was confirmed (*P* for trend < 0.001). In addition to grip strength, single-leg balance was also negatively associated with the incidence of diabetes, even after adjustment for potential confounders, including BMI (*P* < 0.001). On the other hand, for relative vertical jump and trunk flexibility, although the lower quartiles were associated with a higher risk of diabetes in model 1, these negative associations disappeared when BMI was considered. Whole-body reaction time and legs-up were not associated with the incidence of diabetes. We obtained similar results when we performed a sex-stratified analysis (eTable 2 and eTable 3).

**Table 2.** Odds ratios of the incidence of type 2 diabetes mellitus according to quartiles of each physical fitness (*n* = 21,802)

	Quartiles				*P for trend*

	Q4	Q3	Q2	Q1	
Relative grip strength, kg/kg^1^	0.77 (0.67, 0.81)	0.69 (0.57, 0.72)	0.63 (0.51, 0.65)	0.53 (0.44, 0.58)	
Number	5,447	5,453	5,456	5,446	
Panel samples	29,097	28,909	28,517	28,019	
Case	109	215	270	387	
Age- and sex-adjusted OR (95% CI)	1.00 (Reference)	1.32 (1.03, 1.69)	1.68 (1.33, 2.13)	2.64 (2.14, 3.27)	<0.001
Model 1, OR (95% CI)^2^	1.00 (Reference)	1.22 (0.95, 1.56)	1.48 (1.17, 1.87)	2.17 (1.75, 2.70)	<0.001
Model 2, OR (95% CI)^3^	1.00 (Reference)	1.16 (0.90, 1.49)	1.29 (1.01, 1.65)	1.56 (1.23, 1.98)	<0.001

Relative vertical jump, cm/kg^a^	0.83 (0.77, 0.90)	0.71 (0.66, 0.75)	0.63 (0.58, 0.67)	0.52 (0.47, 0.58)	
Number	5,445	5,456	5,452	5,449	
Panel samples	28,787	28,838	28,787	28,130	
Cases	166	198	246	362	
Age- and sex-adjusted OR (95% CI)	1.00 (Reference)	1.27 (0.99, 1.62)	1.50 (1.21, 1.87)	2.31 (1.87, 2.85)	<0.001
Model 1, OR (95% CI)^b^	1.00 (Reference)	1.16 (0.91, 1.49)	1.29 (1.04, 1.61)	1.83 (1.48, 2.27)	<0.001
Model 2, OR (95% CI)^c^	1.00 (Reference)	1.08 (0.84, 1.39)	1.08 (0.86, 1.37)	1.17 (0.91, 1.49)	0.22

Single-leg balance, s^a^	100.0 (72.0, 140.0)	52.0 (36.0, 65.0)	28.0 (19.0, 36.0)	10.0 (6.0, 15.0)	
Number	5,393	5,348	5,402	5,659	
Panel samples	28,833	28,431	28,272	29,006	
Cases	181	210	294	287	
Age- and sex-adjusted OR (95% CI)	1.00 (Reference)	1.14 (0.91, 1.43)	1.73 (1.39, 2.14)	1.73 (1.41, 2.12)	<0.001
Model 1, OR (95% CI)^b^	1.00 (Reference)	1.09 (0.87, 1.36)	1.60 (1.29, 1.98)	1.50 (1.26, 1.90)	<0.001
Model 2, OR (95% CI)^c^	1.00 (Reference)	1.03 (0.83, 1.29)	1.49 (1.20, 1.85)	1.39 (1.13, 1.71)	<0.001

Trunk flexibility, cm^a^	17.0 (14.0, 19.0)	10.0 (8.0, 13.0)	5.0 (3.0, 9.0)	−1.0 (−6.0, 1.0)	
Number	5,011	5,303	5,628	5,860	
Panel samples	26,613	28,213	29,438	30,273	
Cases	209	213	261	289	
Age- and sex-adjusted OR (95% CI)	1.00 (Reference)	1.13 (0.93, 1.38)	1.22 (1.00, 1.49)	1.31 (1.08, 1.59)	0.005
Model 1, OR (95% CI)^b^	1.00 (Reference)	1.07 (0.88, 1.31)	1.14 (0.93, 1.39)	1.20 (0.99, 1.46)	0.019
Model 2, OR (95% CI)^c^	1.00 (Reference)	1.03 (0.84, 1.25)	1.08 (0.89, 1.32)	1.10 (0.91, 1.34)	0.23

Whole-body reaction time, s^a^	410.0 (386.0, 442.0)	362.0 (349.0, 379.0)	335.0 (326.0, 349.0)	306.0 (294.0, 316.0)	
Number	5,523	5,247	5,731	5,301	
Panel samples	28,127	27,471	30,311	28,633	
Cases	249	240	260	223	
Age- and sex-adjusted OR (95% CI)	1.00 (Reference)	0.95 (0.78, 1.16)	0.89 (0.73, 1.09)	0.98 (0.79, 1.20)	0.68
Model 1, OR (95% CI)^b^	1.00 (Reference)	0.95 (0.78, 1.16)	0.90 (0.73, 1.11)	1.01 (0.82, 1.24)	0.92
Model 2, OR (95% CI)^c^	1.00 (Reference)	0.98 (0.80, 1.19)	0.94 (0.77, 1.15)	1.06 (0.87, 1.31)	0.67

Legs-up, s^a,d^	90.0 (90.0, 90.0)	58.0 (42.0, 68.0)			
Number	15,102	6,700			
Panel samples	79,433	35,109			
Cases	756	226			
Age- and sex-adjusted OR (95% CI)	1.00 (Reference)	1.26 (1.08, 1.46)			
Model 1, OR (95% CI)^b^	1.00 (Reference)	1.00 (0.85, 1.17)			
Model 2, OR (95% CI)^c^	1.00 (Reference)	0.98 (0.83, 1.15)			

CI, confidential interval; OR, odds ratio.

^a^Values are represented as medians (interquartile ranges) at baseline.

^b^Adjusted for age (continuous variable), sex (man or woman), smoking status (never smoker, former smoker, or current smoker), drinking status (none, 1–3 days per week, 4–6 days per week, or 7 days per week), breakfast skipping (no or yes), hypertension (no or yes), and dyslipidemia (no or yes).

^c^Variables in model 1 plus body mass index (<18.5, ≥18.5 and <25.0, ≥25.0 and <30.0, or ≥30).

^d^Because 69.3% of participants could keep their legs up for 90 s, this variable was categorized into 2 groups: <90 seconds or ≥90 s.

Because the interactions of grip strength (*P* = 0.002), vertical jump (*P* = 0.001), and single-leg balance (*P* = 0.004) with age were confirmed, an age-stratified analysis (<50 and ≥50 year) for these three variables was performed using the same model as the aforementioned covariates (Table 3). Participants were stratified based on younger than 50 years and equal to or older than 50 years according to the median age. Although the association between single-leg balance and diabetes among participants ≥50 year old was attenuated, there remained a negative association between these three variables and diabetes incidence. No other interaction between physical fitness and covariates was found.

**Table 3.** Odds ratios of the incidence of type 2 diabetes mellitus according to quartiles of each physical fitness stratified by age

	Quartiles				*P for trend*

	Q4	Q3	Q2	Q1	
Relative grip strength, kg/kg					
Age <50 years					
Number	2,648	2,651	2,653	2,648	
Panel samples	14,507	14,512	14,205	13,907	
Case	29	66	82	164	
Model 1, OR (95% CI)^a^	1.00 (Reference)	1.15 (0.86, 1.54)	1.38 (1.06, 1.79)	1.72 (1.33, 2.22)	<0.001
Model 2, OR (95% CI)^b^	1.00 (Reference)	1.24 (0.77, 1.98)	1.34 (0.85, 2.13)	1.87 (1.21, 2.89)	0.002
Age ≥50 years					
Number	2,799	2,802	2,803	2,978	
Panel samples	14,590	14,397	14,312	14,112	
Case	80	149	188	214	
Model 1, OR (95% CI)^a^	1.00 (Reference)	1.15 (0.86, 1.54)	1.38 (1.06, 1.79)	1.72 (1.33, 2.22)	<0.001
Model 2, OR (95% CI)^b^	1.00 (Reference)	1.13 (0.84, 1.52)	1.29 (0.98, 1.69)	1.45 (1.10, 1.91)	0.004

Relative vertical jump, cm/kg					
Age <50 years					
Number	2,647	2,653	2,652	2,648	
Panel samples	14,351	14,344	14,423	14,013	
Case	35	56	76	174	
Model 1, OR (95% CI)^a^	1.00 (Reference)	1.30 (0.80, 2.11)	1.53 (1.01, 2.32)	3.05 (2.04, 4.55)	<0.001
Model 2, OR (95% CI)^b^	1.00 (Reference)	1.13 (0.69, 1.86)	1.10 (0.71, 1.71)	1.37 (0.86, 2.91)	0.25
Age ≥50 years					
Number	2,798	2,803	2,800	2,801	
Panel samples	14,436	14,494	14,364	14,117	
Case	131	142	170	188	
Model 1, OR (95% CI)^a^	1.00 (Reference)	1.13 (0.85, 1.50)	1.23 (0.94, 1.60)	1.44 (1.11, 1.86)	0.003
Model 2, OR (95% CI)^b^	1.00 (Reference)	1.08 (0.81, 1.45)	1.11 (0.84, 1.47)	1.12 (0.84, 1.50)	0.54

Single-leg balance, s					
Age <50 year					
Number	2,616	2,646	2,599	2,739	
Panel samples	14,316	14,388	13,989	14,438	
Case	49	69	110	113	
Model 1, OR (95% CI)^a^	1.00 (Reference)	1.33 (0.84, 2.10)	2.23 (1.47, 3.39)	2.37 (1.57, 3.57)	<0.001
Model 2, OR (95% CI)^b^	1.00 (Reference)	1.18 (0.75, 1.87)	1.90 (1.24, 2.89)	1.91 (1.26, 2.90)	0.005
Age ≥50 year					
Number	2,777	2,702	2,803	2,920	
Panel samples	14,517	14,043	14,283	14,568	
Case	132	141	184	174	
Model 1, OR (95% CI)^a^	1.00 (Reference)	1.01 (0.76, 1.34)	1.39 (1.09, 1.78)	1.30 (1.01, 1.66)	0.006
Model 2, OR (95% CI)^b^	1.00 (Reference)	0.99 (0.74, 1.31)	1.35 (1.05, 1.72)	1.23 (0.96, 1.58)	0.022

CI, confidential interval; OR, odds ratio.

^a^Adjusted for age (continuous variable), sex (man or woman), smoking status (never smoker, former smoker, or current smoker), drinking status (none, 1–3 days per week, 4–6 days per week, or 7 days per week), breakfast skipping (no or yes), hypertension (no or yes), and dyslipidemia (no or yes).

^b^Variables in model 1 plus body mass index (<18.5, ≥18.5 and <25.0, ≥25.0 and <30.0, or ≥30).

### Sensitivity analysis

To minimize the influence of preexisting diabetes at baseline on the relationship between physical fitness and the incidence of T2DM, we performed a sensitivity analysis that excluded participants who developed diabetes within 2 year after the start of follow-up (eTable 4). We confirmed negative relationships of relative grip strength and one-leg balance with the incidence of diabetes. In addition, the complete-case analyses also showed comparable results with analyses using the imputed dataset, even when 1-year lagged exposure and covariates were considered (eTable 5 and eTable 6). For grip strength, absolute grip strength was negatively associated with the incidence of T2DM only when BMI was considered (eTable 7). Finally, the relationship of relative grip strength and single-leg balance with the incidence of diabetes was confirmed when considering other physical fitness performance results, respectively (eTable 8).

## DISCUSSION

We conducted a longitudinal study to examine the association between various components of physical fitness and the incidence of T2DM among Japanese adults. There were negative dose-response relationships between relative grip strength and single-leg balance, which are regarded as indices of muscle strength and static balance, respectively, and the incidence of T2DM.

Skeletal muscle is essential for body movement and has also been identified as the major tissue in glucose metabolism. Muscle strength is defined as the ability of muscle to exert force. Given that the contractions of muscle allow us to conduct all our voluntary activities, including work, leisure, and activities of daily living, muscle strength is considered as the most fundamental component of fitness. Because muscle strength may be improved not only by muscle-strengthening activities, such as resistance training, but also by physical activity intervention^31^^,^^32^—both having favorable effects on the prevention of T2DM^33^—muscle strength may be a predictor of the incidence of T2DM. Some previous studies have reported that a higher grip strength (an indicator of overall muscle strength) at baseline was associated with a lower subsequent risk of T2DM.^8^^,^^9^ However, this association was not always confirmed.^10^^–^^13^ In this study, we obtained a negative dose-response relationship between the time-varying quartiles of relative grip strength, defined by grip strength divided by body weight, and the risk of T2DM.

One of the explanations for the inconsistency between our finding and previous studies may be the difference in the calculation of grip strength. Previous studies used grip strength as an absolute values,^10^^–^^12^ whereas this study used grip strength as a value relative to body weight, because grip strength is influenced by body size.^19^^,^^20^ Interestingly, a negative relationship between absolute grip strength and diabetes appeared when BMI was considered.^11^^,^^12^ In this study, although absolute grip strength was not associated with the risk of diabetes when BMI was not considered, we found a negative relationship when BMI was considered. In addition, a recent study reported that grip strength was not associated with the incidence of T2DM when two major risk scores of T2DM were considered.^13^ The risk scores include several important factors, such as waist circumference, heart rate, diet, and physical activity, which were not considered in this study. Therefore, the relationship of relative grip strength with the incidence of T2DM may be attenuated when these factors are considered.

Because muscle power is also a muscular component of fitness and has many determinants in common with grip strength, it is easy to assume that muscle power also associated with the risk of T2DM. Indeed, relative vertical jump was strongly correlated with relative grip strength in this study (*r* = 0.62). Nevertheless, relative vertical jump was not associated with the risk of diabetes when BMI was considered. This is because that vertical jump is determined by not only strength but also speed.^14^ A previous study showed that, even though obese individuals developed higher muscle strength than normal-weight individuals, the power measured by a jump test in obese individuals was similar to that in normal-weight individuals.^34^ This result supported the possibility that power, particularly the speed component, may be more sensitive to the influence of BMI than muscle strength. Indeed, the correlation of relative vertical jump (*r* = −0.54) with BMI was stronger than that of relative grip strength in this study (*r* = −0.32).

In addition to power, although muscle endurance also shares characteristics in common with strength, this component refer to the ability to perform a specific muscular action for a prolonged period of time, not just a bout. Katzmarzyk et al reported that higher muscle endurance was associated with a lower risk of T2DM in the Canadian Physical Activity Longitudinal Study.^10^ Contrary to these findings, we confirmed no association between muscle endurance and T2DM. One of the reasons for this discrepancy may be because of methodological difference. Katzmarzyk et al used the number of push-ups without time limit as the measure of muscle endurance, whereas we used legs-up test with time limit. Given that about 70% of participants could keep their legs up until the limit, our test could not have stratified our participants, especially participants with higher muscle endurance. Because our participants largely consisted of middle-aged people, the task was easy to complete for them.

Interestingly, we found a negative relationship between single-leg balance with eyes closed and the incidence of T2DM. To our best knowledge, this study is the first study to examine the association between static balance and the risk of T2DM. One of the potential explanations may be the influence of muscle strength as a confounding factor, because similar neurophysiological mechanisms, such as activation of corticospinal pathways, are involved in the regulation of balance and strength.^35^ In this study, however, the negative association was obtained, even after the adjustment for other physical fitness components, including relative grip strength and vertical jump. This result implies that balance and muscle strength are independent of each other and that balance may have mechanisms different from muscle strength on the incidence of T2DM. Although our data cannot provide further explanations, this interpretation is supported by a small-sized association between balance and lower-extremity muscle strength.^36^ Further studies are needed to accumulate findings on the association between balance and the incidence of T2DM.

Whole-body reaction time is the ability to respond quickly to a stimulus, and depends on several factors, including perception, processing, and response. Therefore, reaction time reflects both neural and muscle contraction processes. In this study, reaction time was not associated with the risk of T2DM. A systematic review reported that the influence of physical activity and exercise including cardiorespiratory, strength, or multicomponent intervention on reaction times was inconsistent.^37^ Given that reaction time is greatly influenced by heredity,^14^ daily physical activity might not sufficiently improve reaction time.

In this study, although forward bend reflecting trunk flexibility showed an inverse association with the risk of T2DM, this association was substantially attenuated when BMI was considered. This finding is consistent with a previous study.^10^ This result suggested that BMI confounded the association between forward bend and diabetes. Individuals with a higher BMI may not have bent their trunk owing to abdominal obesity.

There are several limitations in this study. For the measurement of physical fitness, we did not consider the influence of cardiorespiratory fitness on the relationship between other physical fitness components and diabetes. Given that cardiorespiratory fitness among physical fitness components has the strongest influence on the incidence of diabetes among Japanese men,^38^ the relationship of grip strength and single-leg balance with the risk of diabetes might be attenuated when considering cardiorespiratory fitness. In addition, although we considered several potential confounding factors, we did not rule out the influence of dietary nutrients, as mentioned before. Some studies have shown that dietary factors are related to the risk of diabetes.^39^^,^^40^ It is possible that high-fit individuals have healthier dietary nutrient than low-fit individuals. More importantly, we also could not consider the influence of physical activity, because habitual physical activity had a large number of missing data (45.9%). Therefore, the relationship of grip strength and single-leg balance with the incidence of T2DM may have been overestimated. Finally, only 39.4% of all participants who underwent the health examination at baseline were included in the analyses. This exclusion could possibly have result in substantial selection bias, although the difference in baseline characteristics between the included and excluded participants was not large.

In conclusion, lower performance on the single-leg balance test and lower performance on the grip strength test were associated with a higher risk of T2DM among Japanese.

## APPENDIX A. SUPPLEMENTARY DATA

eMaterial 1. Supplemental methods

eTable 1. Difference of baseline characteristics among the included and excluded participants

eTable 2. Odds ratios of the incidence of type 2 diabetes mellitus according to quartiles of each physical fitness among men (n = 15,153)

eTable 3. Odds ratios of the incidence of type 2 diabetes mellitus according to quartiles of each physical fitness among women (n = 6,649)

eTable 4. Odds ratios of the incidence of type 2 diabetes mellitus according to quartiles of physical fitness excluding those who developed type 2 diabetes within 2-year after the onset of follow up (n = 21,368)

eTable 5. Odds ratios of the incidence of type 2 diabetes mellitus according to quartiles of physical fitness in complete-cases analysis

eTable 6. Odds ratios of the incidence of type 2 diabetes mellitus according to quartiles of 1-year lagged physical fitness in complete-cases analysis

eTable 7. Odds ratios of the incidence of type 2 diabetes mellitus according to quartiles of absolute grip strength

eTable 8. Odds ratios of the incidence of type 2 diabetes mellitus according to quartiles of each physical fitness considering mutual physical fitness
