# Supplementary material for: Physical Fitness Tests and Type 2 Diabetes Among Japanese: A Longitudinal Study From the Niigata Wellness Study
Source: J Epidemiol. 2019 Apr 5;29(4):139–46. doi: 10.2188/jea.JE20170280 (PMC6414803; doi:10.2188/jea.JE20170280)
Supplement: Supplementary file 1 [file je-29-139-s001.pdf]

eMaterial 1. Supplemental methods

Physical fitness tests

Relative grip strength

Muscle strength was measured using a grip strength dynamometer (T.K.K. 5401; Takei Scientific Instruments Co., Ltd, Niigata, Japan) with the individuals in a standing position. The dynamometer was adjusted individually to fit hand size. The individuals were told to keep the dynamometer away from any part of the body, and encouraged to exert maximal grip effort. Grip strength was measured once for each hand alternately. The highest value was used and the relative grip strength (grip strength [kg]/body weight [kg]) was calculated, because grip strength is influenced by body size.[19,20]

Relative vertical jump

Lower extremity muscle power was assessed using a vertical jump-measuring instrument (Jump-DF T.K.K. 5414; Takei Scientific Instruments), by measuring the time that the individual had both feet off the ground. The individuals stood with feet and toes on top of the measurement mat, and performed two vertical jumps with one minute allowed for recovery between attempts. Each individual performed 2 trials, and the best performance was used. Because the vertical jump is also influenced by body size, we calculated relative vertical jump (vertical jump [cm]/body weigh [kg]).

Single-leg balance

Static balance was assessed by measuring the duration (s) of single-leg balance with eyes closed using a stopwatch. Participants chose their lifting leg and were asked to keep standing on a firm surface for 240 s with their hands placed on their hips during the balance test. Participants performed the test a maximum of 3 trials, and the best value was used. The test ended when the eyes opened, the lifting leg touched the ground, the hands departed from their hip, the pivoting foot moved, or the participants reached the goal of 240 s. Although participants basically underwent a single trial, a maximum of two additional trails was allowed for the participants who lost their balance immediately after the start of a trial. If the participants reached the goal of 240 s, no further trials were conducted, and the best value was used. A longer duration represents a better performance of static balance.

Trunk flexibility

Trunk flexibility was measured using a digital flexibility testing device (T.K.K. 5403; Takei Scientific Instruments). Participants were asked to stand on a measuring bench, placing the toes even with the front edge of the bench, and then asked to bend over and reach down as far as possible without bouncing, while keeping the knees locked. Performance was scored as the

distance (cm) reached by the middle fingers. Participants performed a single trial of the flexibility test. The upward distance from the zero mark at the level of the upper surface of the bench was a negative score and the downward distance was a positive score. Therefore, a lager positive score represents a better performance of trunk flexibility. Participants performed a single trial of the flexibility test.

Whole-body reaction time

Whole-body reaction time was measured using a pressure-sensing mat (T.K.K. 5408; Takei Scientific Instruments). Participants were asked to stand on the mat switch with their knees slightly bent without shoes and to jump upright as quickly as possible in response to a light sign that was 2 m away from the mat. The time (ms) between the flashing and the disappearance of foot pressure from the mat was measured. Participants underwent 3 trials and the average of the 3 trials was calculated. The test was re-performed when participants passed over the flashing or reacted before the flashing. A shorter reaction time represents a better performance of whole-body reaction time.

Lower abdominal muscular endurance

A supine legs-up test was used to measure the muscular endurance of the lower abdominal muscles. The participants began in the spine-lying position, hip and legs extended, with the hands placing beside or on the trunk, or crossing under their head. Next, the participants were asked to raise both extended legs from the floor approximately 30 cm and hold this position as long as possible without any tilting in the pelvis. The time (s) was recorded by a stopwatch and the test was ended when the participants were no longer able to maintain legs clearance. Participants performed a single trial of the legs-up test. Participants performed a single trial of the legs-up test. A longer time represents a better performance of muscular endurance of lower abdominal muscles.

REFERENCES

19. Kawamoto R, Ninomiya D, Kasai Y, Kusunoki T, Ohtsuka N, Kumagi T, et al. Handgrip strength is associated with metabolic syndrome among middle-aged and elderly community-dwelling persons. Clin Exp Hypertens. 2016;38:245-251.
20. Ramirez-Velez R, Correa-Bautista JE, Lobelo F, Izquierdo M, Alonso-Martinez A, Rodriguez-Rodriguez F, et al. High muscular fitness has a powerful protective cardiometabolic effect in adults: influence of weight status. BMC Public Health. 2016;16:1012.

eTable 1. Difference of baseline characteristics among the included and excluded participants

	Excluded (n=33,545)	Included (n=21,802)
Women, n (%)	12,728 (37.9)	6,649 (30.5)
Age, years	52.0 (46.0, 59.0)	50.0 (44.0, 56.0)
Height, cm	163.1 (156.3, 169.2)	164.9 (158.2, 170.6)
Weight, kg	60.2 (53.1, 68.1)	61.4 (54.2, 68.5)
BMI, kg/m^2	22.8 (20.8, 24.8)	22.7 (20.9, 24.5)
SBP, mm Hg	119.0 (109.0, 130.0)	117.0 (108.0, 127.0)
DBP, mm Hg	77.0 (68.0, 84.0)	76.0 (68.0, 83.0)
TG, mg/dL	97.0 (68.0, 143)	96.0 (67.0, 139.0)
LDL-C, mg/dL	118.0 (98.0, 140.0)	117.0 (98.0, 139.0)
HDL-C, mg/dL	59.0 (49.0, 70.0)	59.0 (50.0, 71.0)
TC, mg/dL	203.0 (181.0, 226.0)	202.0 (181.0, 224.0)
Blood glucose, mg/dL	95.0 (89.0, 104.0)	93.0 (88.0, 100.0)
HbA1c, mmol/mol	33.3 (31.2, 36.6)	32.2 (30.1, 35.5)
HbA1c (%)	5.2 (5.0, 5.5)	5.1 (4.9, 5.4)
Smoking status, n (%)		
Never smoker	15,651 (46.7)	10,034 (46.0)
Former smoker	5,473 (16.3)	4,290 (19.7)
Current smoker	12,360 (36.8)	7,416 (34.0)
Missing data	61 (0.2)	62 (0.3)
Drinking status, n (%)		
None	11,044 (32.9)	5,496 (25.2)
1-2 days/week	4,827 (14.4)	3,442 (15.8)
3-6 days/week	6,623 (19.7)	5,099 (23.4)
7 days/week	10,987 (32.8)	7,704 (35.3)
Missing data	64 (0.2)	61 (0.3)
Skipping breakfast, n (%)		
No	31,082 (92.7)	20,470 (93.9)
Yes	2,401 (7.2)	1,271 (5.8)
Missing data	62 (0.2)	61 (0.3)
Exercise habit, n (%)		
No	16,024 (47.8)	9,497 (43.6)
Yes	7,730 (23.0)	6,255 (28.7)
Missing data	9,791 (29.2)	6,050 (27.7)
Hypertension, n (%)	8,671 (25.8)	4,263 (19.6)
Dyslipidemia, n (%)	14,634 (43.6)	9,130 (41.9)
Relative grip strength, kg/kg	0.60 (0.51, 0.69)	0.63 (0.54, 0.71)
Missing data, n (%)	14,104 (42.0)	

Relative vertical jump, cm/kg	0.64 (0.54, 0.73)	0.66 (0.57, 0.76)
Missing data, n (%)	19,581 (58.4)	
Single-leg balance, s	24.0 (11.0, 52.0)	35.0 (16.0, 66.0)
Missing data, n (%)	18,338 (54.7)	
Forward bend, cm	7.0 (2.0, 13.0)	8.0 (3.0, 13.0)
Missing data, n (%)	18,505 (55.2)	
Reaction time, ms	360.0 (331.0, 398.0)	349.0 (323.0, 383.0)
Missing data, n (%)	18,430 (54.9)	
Legs-up, s	90 (63.0, 90.0)	90.0 (72.0, 90.0)
Missing data, n (%)	20,008 (59.6)	
Diabetes incidence, n (%)	1,057 (3.5)	972 (4.5)

BMI, body mass index, calculated as weight in kilograms divided by height in meters squared; DBP, diastolic blood pressure; HbA1c, hemoglobin A1c; HDL-C, high-density lipoprotein cholesterol; IQR, interquartile range; LDL-C, low-density lipoprotein cholesterol; SBP, systolic blood pressure; TC, total cholesterol; TG, triglyceride.

Data are expressed as medians (interquartile range) for continuous variable and number (percentage) for categorical variable.

eTable 2. Odds ratios of the incidence of type 2 diabetes mellitus according to quartiles of each physical fitness among men (n=15,153)

	Quartiles				
	Q4	Q3	Q2	Q1	*P for trend*
Relative grip strength, kg/kg[a]	0.79 (0.76, 0.83)	0.71 (0.69, 0.73)	0.64 (0.62, 0.66)	0.56 (0.53, 0.59)	
Number	3,785	3,791	3,794	3,783	
Panel samples	20,353	20,209	19,990	19,454	
Case	91	174	224	307	
Age-adjusted OR (95% CI)	1.00 (Reference)	1.34 (1.03, 1,75)	1.78 (1.35, 2.34)	2.69 (2.13, 3.41)	<0.001
Model 1, OR (95% CI)[b]	1.00 (Reference)	1.23 (0.94, 1.60)	1.55 (1.17, 2.05)	2.19 (1.72, 2.79)	<0.001
Model 2, OR (95% CI)[c]	1.00 (Reference)	1.15 (0.88, 1.51)	1.33 (1.00, 1.77)	1.54 (1.19, 2.01)	0.001
Relative vertical jump, cm/kg[a]	0.85 (0.80, 0.92)	0.73 (0.69, 0.76)	0.65 (0.62, 0.68)	0.55 (0.50, 0.59)	
Number	3,786	3,790	3,791	3,786	
Panel samples	20,110	20,126	20,149	19,621	
Cases	140	167	201	288	
Age-adjusted OR (95% CI)	1.00 (Reference)	1.32 (1.01, 1.71)	1.45 (1.14, 1.84)	2.25 (1.80, 2.81)	<0.001
Model 1, OR (95% CI)[b]	1.00 (Reference)	1.20 (0.93, 1.56)	1.24 (0.97, 1.57)	1.78 (1.42, 2.23)	<0.001
Model 2, OR (95% CI)[c]	1.00 (Reference)	1.10 (0.85, 1.44)	1.01 (0.78, 1.30)	1.09 (0.84, 1.42)	0.67
Single-leg balance, s[a]	95.0 (69.0, 132.0)	50.0 (36.0, 61.0)	26.0 (18.0, 34.0)	10.0 (6.0, 15.0)	
Number	3,744	3,721	3,751	3,937	
Panel samples	20,150	19,743	19,684	20,429	
Cases	154	168	243	231	
Age-adjusted OR (95% CI)	1.00 (Reference)	1.20 (0.95, 1.53)	1.78 (1.41, 2.25)	1.73 (1.38, 2.17)	<0.001
Model 1, OR (95% CI)[b]	1.00 (Reference)	1.15 (0.90, 1.46)	1.65 (1.30, 2.09)	1.57 (1.25, 1.97)	<0.001

Model 2, OR (95% CI)[c]	1.00 (Reference)	1.09 (0.86, 1.38)	1.53 (1.21, 1.94)	1.41 (1.13, 1.78)	<0.001
Trunk flexibility, cm[a]	15.0 (13.0, 17,0)	9.0 (8.0, 10.0)	4.0 (3.0, 5.0)	-3.0 (-7.0, 0.0)	
Number	3,517	3,695	3,895	4,046	
Panel samples	18,708	19,750	20,524	21,024	
Cases	173	171	216	236	
Age-adjusted OR (95% CI)	1.00 (Reference)	1.11 (0.88, 1.40)	1.21 (0.97, 1.52)	1.31 (1.06, 1.62)	0.009
Model 1, OR (95% CI)[b]	1.00 (Reference)	1.06 (0.84, 1.33)	1.13 (0.91, 1.42)	1.21 (0.98, 1.50)	0.062
Model 2, OR (95% CI)[c]	1.00 (Reference)	1.01 (0.80, 1.27)	1.07 (0.86, 1.34)	1.11 (0.89, 1.37)	0.29
Whole-body reaction time, s[a]	397.0 (378.0, 423.0)	354.0 (346.0, 365.0)	330.0 (323.0, 337.0)	301.0 (290.0, 309.0)	
Number	3,752	3,724	4,055	3,622	
Panel samples	19,198	19,608	21,548	19,652	
Cases	205	193	216	182	
Age-adjusted OR (95% CI)	1.00 (Reference)	0.88 (0.71, 1.10)	0.84 (0.67, 1.04)	0.98 (0.79, 1.21)	0.74
Model 1, OR (95% CI)[b]	1.00 (Reference)	0.88 (0.71, 1.10)	0.84 (0.68, 1.05)	1.01 (0.81, 1.25)	0.93
Model 2, OR (95% CI)[c]	1.00 (Reference)	0.90 (0.72, 1.11)	0.87 (0.70, 1.08)	1.05 (0.85, 1.30)	0.74
Legs-up, s[a,d]	90.0 (90.0, 90.0)	62.0 (50.0, 71.0)			
Number	12,014	3,139			
Panel samples	63,417	16,589			
Cases	652	144			
Age-adjusted OR (95% CI)	1.00 (Reference)	1.04 (0.87)			
Model 1, OR (95% CI)[b]	1.00 (Reference)	0.98 (0.82, 1.17)			
Model 2, OR (95% CI)[c]	1.00 (Reference)	0.95 (0.79, 1.14)			

CI, confidential interval; OR, odds ratio.

[a] Values are represented as medians (interquartile ranges) at baseline.

[b] Adjusted for age (continuous variable), smoking status (never smoker, former smoker, or current smoker), drinking status (none, 1-3 days/week, 4-6 days/week, or 7 days/week), breakfast skipping (no or yes), hypertension (no or yes), and dyslipidemia (no or yes).

[c] Variables in model 1 plus body mass index (<18.5, ≥18.5 and <25.0, ≥25.0 and <30.0, or ≥30).

[d] Because 79.3% of participants could keep their legs up for 90 s, this index was categorized into 2 groups: <90 s or ≥90 s.

eTable 3. Odds ratios of the incidence of type 2 diabetes mellitus according to quartiles of each physical fitness among women (n=6,649)

	Quartiles				
	Q4	Q3	Q2	Q1	*P for trend*
Relative grip strength, kg/kg[a]	0.62 (0.59, 0.65)	0.54 (0.52, 0.56)	0.48 (0.46, 0.51)	0.41 (0.38, 0.44)	
Number	1,662	1,662	1,662	1,663	
Panel samples	8,744	8,700	8,527	8,565	
Case	18	41	46	71	
Age-adjusted OR (95% CI)	1.00 (Reference)	1.22 (0.68, 2.20)	1.30 (0.74, 2.26)	2.46 (1.44, 4.21)	<0.001
Model 1, OR (95% CI)[b]	1.00 (Reference)	1.19 (0.66, 2.15)	1.20 (0.69, 2.08)	2.13 (1.23, 3.68)	0.004
Model 2, OR (95% CI)[c]	1.00 (Reference)	1.20 (0.66, 2.17)	1.14 (0.64, 2.01)	1.66 (0.91, 3.03)	0.095
Relative vertical jump, cm/kg[a]	0.77 (0.71, 0.84)	0.65 (0.60, 0.70)	0.57 (0.53, 0.61)	0.47 (0.41, 0.51)	
Number	1,659	1,666	1,661	1,663	
Panel samples	8,677	8,712	8,638	8,509	
Cases	26	31	45	74	
Age-adjusted OR (95% CI)	1.00 (Reference)	1.02 (0.51, 2.06)	1.77 (0.97, 3.23)	2.63 (1.55, 4.46)	<0.001
Model 1, OR (95% CI)[b]	1.00 (Reference)	0.95 (0.47, 1.92)	1.57 (0.86, 2.89)	2.12 (1.24, 3.63)	0.001
Model 2, OR (95% CI)[c]	1.00 (Reference)	0.96 (0.46, 1.99)	1.50 (0.78, 2.89)	1.56 (0.83, 2.95)	0.067
Single-leg balance, s[a]	112.0 (82.0, 162.5)	58.0 (40.0, 71.0)	30.0 (20.0, 39.0)	10.0 (6.0,16.0)	
Number	1,649	1,627	1,651	1,722	
Panel samples	8,683	8,688	8,588	8,577	
Cases	27	42	51	56	
Age-adjusted OR (95% CI)	1.00 (Reference)	0.89 (0.50, 1.58)	1.52 (0.94, 2.46)	1.71 (1.10, 2.67)	0.003
Model 1, OR (95% CI)[b]	1.00 (Reference)	0.85 (0.48, 1.51)	1.40 (0.87, 2.27)	1.47 (0.94, 2.32)	0.025

Model 2, OR (95% CI)[c]	1.00 (Reference)	0.82 (0.46, 1.45)	1.31 (0.81, 2.12)	1.28 (0.81, 2.03)	0.110
Trunk flexibility, cm[a]	19.0 (18.0, 21.0)	15.0 (14.0, 16.0)	11.0 (9.0, 12.0)	4.0 (1.0, 6.0)	
Number	1494	1,608	1,733	1,814	
Panel samples	7905	8,463	8,914	9,254	
Cases	36	42	45	53	
Age-adjusted OR (95% CI)	1.00 (Reference)	1.14 (0.71, 1.83)	1.16 (0.75, 1.79)	1.17 (0.74, 1.86)	0.49
Model 1, OR (95% CI)[b]	1.00 (Reference)	1.14 (0.71, 1.83)	1.15 (0.75, 1.78)	1.16 (0.74, 1.83)	0.52
Model 2, OR (95% CI)[c]	1.00 (Reference)	1.11 (0.69, 1.77)	1.10 (0.72, 1.70)	1.09 (0.69, 1.71)	0.74
Whole-body reaction time, s[a]	437.0 (415.0, 467.0)	385.0 (376.0, 395.0)	355.0 (347.0, 363.0)	323.0 (310.0, 332.0)	
Number	1711	1,523	1,676	1,679	
Panel samples	8929	7,863	8,763	8,981	
Cases	44	47	44	41	
Age-adjusted OR (95% CI)	1.00 (Reference)	1.30 (0.80, 2.11)	1.18 (0.74, 1.89)	0.94 (0.58, 1.55)	0.73
Model 1, OR (95% CI)[b]	1.00 (Reference)	1.33 (0.82, 2.16)	1.23 (0.77, 1.98)	1.00 (0.61, 1.65)	0.94
Model 2, OR (95% CI)[c]	1.00 (Reference)	1.41 (0.87, 2.30)	1.32 (0.82, 2.13)	1.10 (0.67, 1.82)	0.76
Legs-up, s[a,d]	90.0 (90.0, 90.0)	51.0 (35.0, 64.0)			
Number	3,088	3,561			
Panel samples	16,016	18,520			
Cases	94	82			
Age-adjusted OR (95% CI)	1.00 (Reference)	1.23 (0.86, 1.70)			
Model 1, OR (95% CI)[b]	1.00 (Reference)	1.06 (0.76, 1.47)			
Model 2, OR (95% CI)[c]	1.00 (Reference)	1.06 (0.76, 1.47)			

CI confidential interval; OR, odds ratio.

[a] Values are represented as medians (interquartile ranges) at baseline.

[b] Adjusted for age (continuous variable), smoking status (never smoker, former smoker, or current smoker), drinking status (none, 1-3 days/week, 4-6 days/week, or 7 days/week), breakfast skipping (no or yes), hypertension (no or yes), and dyslipidemia (no or yes).

[c] Variables in model 1 plus body mass index (<18.5, ≥18.5 and <25.0, ≥25.0 and <30.0, or ≥30).

[d] Because 46.4% of participants could keep their legs up for 90 s, this index was categorized into 2 groups: <90 s or ≥90 s.

eTable 4. Odds ratios of the incidence of type 2 diabetes mellitus according to quartiles of physical fitness excluding those who developed type 2 diabetes within 2-year after the onset of follow up (n=21,368)

	Quartiles				
	Q4	Q3	Q2	Q1	*P for trend*
Relative grip strength, kg/kg[a]	0.77 (0.67, 0.81)	0.69 (0.57,0.72)	0.63 (0.51, 0.65)	0.53 (0.44, 0.58)	
Number	5,403	5,375	5,342	5,266	
Panel samples	28,993	28,720	28,247	27,593	
Case	65	137	156	198	
Age- and sex-adjusted OR (95% CI)	1.00 (Reference)	1.32 (1.03, 1.69)	1.68 (1.33, 2.13)	2.64 (2.14, 3.27)	<0.001
Model 1, OR (95% CI)[b]	1.00 (Reference)	1.22 (0.95, 1.56)	1.48 (1.17, 1.87)	2.17 (1.75, 2.70)	<0.001
Model 2, OR (95% CI)[c]	1.00 (Reference)	1.16 (0.90, 1.49)	1.29 (1.01, 1.65)	1.56 (1.23, 1.98)	0.031
Single-leg balance, s[a]	100 (73.0, 141.0)	52.0 (37.0, 65.0)	28.0 (19.0, 36.0)	10.0 (6.0, 15.0)	
Number	5,316	5,267	5,275	5,528	
Panel samples	28,648	28,242	27,973	28,690	
Cases	104	129	167	156	
Age- and sex-adjusted OR (95% CI)	1.00 (Reference)	1.14 (0.91, 1.43)	1.73 (1.39, 2.14)	1.73 (1.41, 2.12)	<0.001
Model 1, OR (95% CI)[b]	1.00 (Reference)	1.09 (0.87, 1.36)	1.60 (1.39, 1.98)	1.55 (1.26, 1.90)	0.001
Model 2, OR (95% CI)[c]	1.00 (Reference)	1.03 (0.83, 1.29)	1.49 (1.20, 1.85)	1.39 (1.13, 1.71)	0.015

CI, confidential interval; OR, odds ratio.

[a] Values are represented as medians (interquartile ranges) at baseline.

[b] Adjusted for age (continuous variable), sex (men or women), smoking status (never smoker, former smoker, or current smoker), drinking status (none, 1-3 days/week, 4-6 days/week, or 7 days/week), breakfast skipping (no or yes), hypertension (no or yes), and dyslipidemia (no or yes).

[c] Variables in model 1 plus body mass index (<18.5, ≥18.5 and <25.0, ≥25.0 and <30.0, or ≥30).

eTable 5. Odds ratios of the incidence of type 2 diabetes mellitus according to quartiles of physical fitness in complete-cases analysis

	Quartiles				
	Q4	Q3	Q2	Q1	*P for trend*
Relative grip strength, kg/kg[a]	0.77 (0.72, 0.82)	0.70 (0.64, 0.72)	0.64 (0.57, 0.66)	0.55 (0.47, 0.59)	
Number	1,334	1,341	1,343	1,335	
Panel samples	9,240	9,253	9,246	9,026	
Case	35	59	57	112	
Age- and sex-adjusted OR (95% CI)	1.00 (Reference)	1.57 (1.04, 2.37)	1.38 (0.90, 2.10)	3.19 (2.20, 4.62)	<0.001
Model 1, OR (95% CI)[b]	1.00 (Reference)	1.44 (0.95, 2.18)	1.20 (0.78, 1.84)	2.55 (1.75, 3.72)	<0.001
Model 2, OR (95% CI)[c]	1.00 (Reference)	1.38 (0.90, 2.10)	1.08 (0.69, 1.67)	1.93 (1.27, 2.92)	0.004
Single-leg balance, s[a]	111.0 (85.0, 150.3)	59.0 (45.0, 71.0)	33.0 (24.0, 41.0)	13.0 (7.0, 19.0)	
Number	1,178	1,189	1,194	1,214	
Panel samples	8,150	8,188	8,167	8,301	
Cases	33	51	69	73	
Age- and sex-adjusted OR (95% CI)	1.00 (Reference)	1.11 (0.71, 1.72)	1.66 (1.11, 2.48)	2.09 (1.42, 3.07)	<0.001
Model 1, OR (95% CI)[b]	1.00 (Reference)	1.02 (0.66, 1.59)	1.49 (0.99, 2.22)	1.83 (1.24, 2.70)	<0.001
Model 2, OR (95% CI)[c]	1.00 (Reference)	0.96 (0.62, 1.49)	1.37 (0.91, 2.06)	1.63 (1.10, 2.41)	0.002

CI, confidential interval; OR, odds ratio.

[a] Values are represented as medians (interquartile ranges) at baseline.

[b] Adjusted for age (continuous variable), sex (man or woman), smoking status (never smoker, former smoker, or current smoker), drinking status (none, 1-3 days/week, 4-6 days/week, or 7 days/week), breakfast skipping (no or yes), hypertension (no or yes), and dyslipidemia (no or yes).

[c] Variables in model 1 plus body mass index (<18.5, ≥18.5 and <25.0, ≥25.0 and <30.0, or ≥30).

eTable 6. Odds ratios of the incidence of type 2 diabetes mellitus according to quartiles of 1-year lagged physical fitness in complete-cases analysis

	Quartiles				
	Q4	Q3	Q2	Q1	*P for trend*
Relative grip strength, kg/kg[a]	0.77 (0.72, 0.82)	0.70 (0.64, 0.72)	0.64 (0.57, 0.66)	0.55 (0.47, 0.59)	
Number	1334	1341	1343	1335	
Panel samples	9240	9253	9246	9026	
Case	35	59	57	112	
Age- and sex-adjusted OR (95% CI)	1.00 (Reference)	1.13 (0.73, 1.75)	1.77 (1.19, 2.64)	3.09 (2.14, 4.46)	<0.001
Model 1, OR (95% CI)[b]	1.00 (Reference)	1.06 (0.69, 1.65)	1.59 (1.06, 2.37)	2.57 (1.77, 3.74)	<0.001
Model 2, OR (95% CI)[c]	1.00 (Reference)	1.00 (0.64, 1.57)	1.40 (0.92, 2.12)	1.87 (1.24, 2.83)	<0.001
Single-leg balance, s[a]	111.0 (85.0, 150.3)	59.0 (45.0, 71.0)	33.0 (24.0, 41.0)	13.0 (7.0, 19.0)	
Number	1178	1189	1194	1214	
Panel samples	8150	8188	8167	8301	
Cases	33	51	69	73	
Age- and sex-adjusted OR (95% CI)	1.00 (Reference)	1.60 (1.04, 2.46)	1.90 (1.25, 2.88)	2.09 (1.39, 3.15)	<0.001
Model 1, OR (95% CI)[b]	1.00 (Reference)	1.48 (0.97, 2.29)	1.72 (1.13, 2.61)	1.84 (1.22, 2.77)	0.004
Model 2, OR (95% CI)[c]	1.00 (Reference)	1.39 (0.90, 2.14)	1.56 (1.03, 2.38)	1.62 (1.07, 2.25)	0.024

CI, confidential interval; OR, odds ratio.

[a] Values are represented as medians (interquartile ranges) at baseline.

[b] Adjusted for age (continuous variable), smoking status (never smoker, former smoker, or current smoker), drinking status (none, 1-3 days/week, 4-6 days/week, or 7 days/week), breakfast skipping (no or yes), hypertension (no or yes), and dyslipidemia (no or yes).

[c] Variables in model 1 plus body mass index (<18.5, ≥18.5 and <25.0, ≥25.0 and <30.0, or ≥30).

eTable 7. Odds ratios of the incidence of type 2 diabetes mellitus according to quartiles of absolute grip strength

	Quartiles				
	Q4	Q3	Q2	Q1	*P for trend*
Absolute grip strength, kg[a]	50.0 (36.0, 53.0)	45.0 (30.0, 48.0)	41.0 (28.0, 44.0)	35.0 (24.0, 39.0)	
Number	4,838	5,405	5,398	6,167	
Panel samples	25,846	28,373	28,521	32,002	
Case	206	222	259	285	
Age- and sex-adjusted OR (95% CI)	1.00 (Reference)	0.99 (0.80, 1.23)	1.02 (0.83, 1.25)	1.06 (0.87, 1.29)	0.51
Model 1, OR (95% CI)[b]	1.00 (Reference)	1.00 (0.81, 1.23)	1.03 (0.84, 1.26)	1.08 (0.88, 1.32)	0.42
Model 2, OR (95% CI)[c]	1.00 (Reference)	1.10 (0.89, 1.36)	1.18 (0.96, 1.45)	1.27 (1.04, 1.57)	0.013

CI, confidential interval; OR, odds ratio.

[a] Values are represented as medians (interquartile ranges) at baseline.

[b] Adjusted for age (continuous variable), smoking status (never smoker, former smoker, or current smoker), drinking status (none, 1-3 days/week, 4-6 days/week, or 7 days/week), breakfast skipping (no or yes), hypertension (no or yes), and dyslipidemia (no or yes).

[c] Variables in model 1 plus body mass index (<18.5, ≥18.5 and <25.0, ≥25.0 and <30.0, or ≥30).

eTable 8. Odds ratios of the incidence of type 2 diabetes mellitus according to quartiles of each physical fitness considering mutual physical fitness

	Quartiles				
	Q4	Q3	Q2	Q1	*P for trend*
Relative grip strength, kg/kg					
Adjusted OR (95% CI)[a]	1.00 (Reference)	1.15 (0.89, 1.48)	1.27 (0.98, 1.63)	1.53 (1.19, 1.98)	<0.001
Single-leg balance, s					
Adjusted OR (95% CI)[a]	1.00 (Reference)	1.03 (0.82, 1.28)	1.48 (1.19, 1.84)	1.37 (1.11, 1.70)	<0.001

CI, confidential interval; OR, odds ratio.

[a] Adjusted for age (continuous variable), sex (man or woman), smoking status (never smoker, former smoker, or current smoker), drinking status (none, 1-3 days/week, 4-6 days/week, or 7 days/week), breakfast skipping (no or yes), hypertension (no or yes), dyslipidemia (no or yes), body mass index (<18.5, ≥18.5 and <25.0, ≥25.0 and <30.0, or ≥30), relative grip strength (for single-leg balance, continuous variable), relative vertical jump (continuous variable), single-leg balance (for relative grip strength, continuous variable), forward bend (continuous variable), reaction time (continuous variable), and legs-up (continuous variable).
